# Hypoxia Enhances Protective Effect of Placental-Derived Mesenchymal Stem Cells on Damaged Intestinal Epithelial Cells by Promoting Secretion of Insulin-Like Growth Factor-1

**DOI:** 10.3390/ijms15021983

**Published:** 2014-01-27

**Authors:** Lili Du, Yanqiu Yu, Haiying Ma, Xiaomei Lu, Ling Ma, Yunan Jin, Haipeng Zhang

**Affiliations:** Department of Pathophysiology, College of Basic Medical Science, China Medical University, Shenyang 110001, China; E-Mails: lilidu1234567@gmail.com (L.D.); yuyanqiu123@gmail.com (Y.Y.); mhysunny@gmail.com (H.M.); xmlu@mail.cmu.edu.cn (X.L.); mlsunny123456@gmail.com (L.M.); jynsunny@gmail.com (Y.J.)

**Keywords:** placental-derived mesenchymal stem cells, caco2, insulin-like growth factor 1, ischemia-reperfusion injury

## Abstract

Apoptosis and necrosis of intestinal epithelial cells (IECs), induced by ischemia-reperfusion (I/R) injury, can lead to dysfunction of the intestinal barrier, which could cause multiple organ dysfunction syndromes. Mesenchymal stem cells (MSCs) have the potential of providing protective effects on damaged IECs via paracrine action. This study investigated whether hypoxia can enhance the protective effect of placental-derived MSCs (pMSCs) on H_2_O_2_-treated-caco2 cells, and explored the possible mechanism. The pMSCs isolated by tissue culture were fibroblast-like, positive for CD73, CD90 and CD105 and can differentiate into chondrocytes and endothelial cells. Five days after treatment with H_2_O_2_, the numbers of living caco2 cells significantly decreased. More live H_2_O_2_-treated-caco2 cells were observed in pMSCs hypoxia culture medium (pMSCs-HCM) than pMSCs normoxia culture medium (pMSCs-NCM), and the application of a specific antibody that blocked insulin-like growth factor-1 (IGF-1) leads to a significant decrease of the protective effect of pMSCs-HCM. Hypoxia can promote IGF-1 expression of pMSCs at mRNA and protein levels, and caco2 stably expressed IGF-1 receptor. Knocking down IGF-1 expression in pMSCs by siRNA resulted in a significant attenuation of the increase in apoptosis of H_2_O_2_-treated-caco2 cultured in pMSCs-HCM. In conclusion, hypoxia can increase the protective effect of pMSCs on H_2_O_2_-treated-caco2 cells via a promotion of their paracrine actions, and the key cytokine involved is IGF-1.

## 1. Introduction

Intestinal ischemia-reperfusion (I/R) injury is a common and serious disease. Intestinal epithelial cells damage can lead to dysfunction of the intestinal barrier and invasion of bacterial toxins, which could further cause multiple organ dysfunction syndromes (MODS) or multiple organ failure (MOF). Currently, prognosis of patients with occlusion of the superior mesenteric artery still remains very poor with mortality rates of such patients between 60% and 100% [1]. Recently, I/R injury is becoming the biggest obstacle to improve the outcome of intestinal transplantation [2]. The mechanisms and treatments for intestinal I/R injury have been explored and investigated in many previous studies; oxygen-free radical formation, inflammatory cytokines, release of iron storage, neutrophils, complement activation, infiltration and enteric bacteria translocation are all thought to be involved [3]. However, treatments for intestinal I/R injury including nitric oxide supplementation, antioxidants, anti-complement therapy, free-radical scavengers, anti-leukocyte therapy, glutamine supplementation and glycine supplementation are still inadequate [4].

Apoptosis and necrosis play key roles in the process of intestinal I/R injury. Under normal circumstances, apoptotic intestinal epithelium cells are limited to the surface and crypt epithelial cells at the base can produce new cells to replace apoptotic cells through regeneration. Apoptosis and regeneration are in a dynamic equilibrium, so the intestinal mucosa is intact. In the pathological case of I/R, the number of dead intestinal epithelial cells increases greatly because of the participation of reactive oxygen and peroxides [5]. Sometimes, intestinal epithelial cell damage may recover, but a critical number of surviving cells is required to reconstitute structural integrity. Some investigations reported that many cytokines played important roles in protection of intestinal epithelial cells [6–8], such as insulin, insulin-like growth factor (IGF), epidermal growth factor and transforming growth factor. Therefore, we supposed that finding a key protective cytokine and supplying more it to the damaged intestinal epithelial cells might be an innovative strategy for the treatment of intestinal I/R injury.

Placental-derived Mesenchymal Stem Cells (pMSCs) are a new kind of stem cell found in recent years. They are pluripotent stem cells derived from the mesoderm in the embryonic development. The pMSCs have similar biological characteristics as bone marrow mesenchymal stem cells: low immunogenicity, the capacity of self-renewal, multipotent differentiation and immunosuppression [9–11]. Additionally, pMSCs also have their own unique advantages: easy to obtain, sufficient number of cells, easy to isolate and culture, and rapid proliferation [12]. They have the stable properties of stem cells, and some studies have shown that they would not form tumors *in vivo* [13,14]. Therefore, it is confirmed that pMSCs are a good source of stem cells. Based on the characteristics of pMSCs, they have a very good therapeutic potential in many diseases, which has been proven by many previous investigations; Pan *et al.* reported that pMSCs can accelerate the repair of sciatic nerve injury [15], Findings of Tonmonori confirmed that transplanted pMSCs can promote the recovery of morphology and function in the injured bladder [16], our study have shown that pMSCs can differentiate into chondrocytes which secrete type II collagen to repair damaged cartilage after two months of transplantation [17]; in a rat model of muscular dystrophy and myocardial infarction, the pMSCs also had therapeutic effect [18,19].

Current studies of stem cell therapy for intestinal I/R injury mainly focus on bone marrow mesenchymal stem cells. Results showed that the beneficial effects of bone marrow mesenchymal stem cells are little mediated via their differentiation into intestinal epithelial cells but rather primarily by secretion of a series of cytokines [20–22]. The pMSCs have not only the similar characteristics with bone marrow mesenchymal stem cells, but also have their own unique advantage (wider origin and easier to obtain). Therefore, if pMSCs also have therapeutic effect on intestinal I/R injury, they represent a better prospect. Additionally, our preliminary results showed that pMSCs culture supernatant could protect intestinal epithelial cells from damage. Some cytokines secreted by pMSCs were closely related to the protective effects. Among these cytokines, insulin-like growth factor-1 (IGF-1) was implicated as an important mediator of protection in a model of intestinal I/R injury [23]. Some other studies showed that hypoxia could enhance the protection of mesenchymal stem cells [24–26], however the mechanisms are still not very clear.

In our present study, we established the damaged caco2 cells *in vitro* model induced by H_2_O_2_ to investigate whether hypoxia could enhance the protective effect of pMSCs on intestinal epithelial cells and explore the possible mechanism. Furthermore, the role of IGF-1 as a key mediator of enhanced protection was also explored in this study.

## 2. Results and Discussion

### 2.1. Isolation and Identification of pMSCs

**Figure 1.**
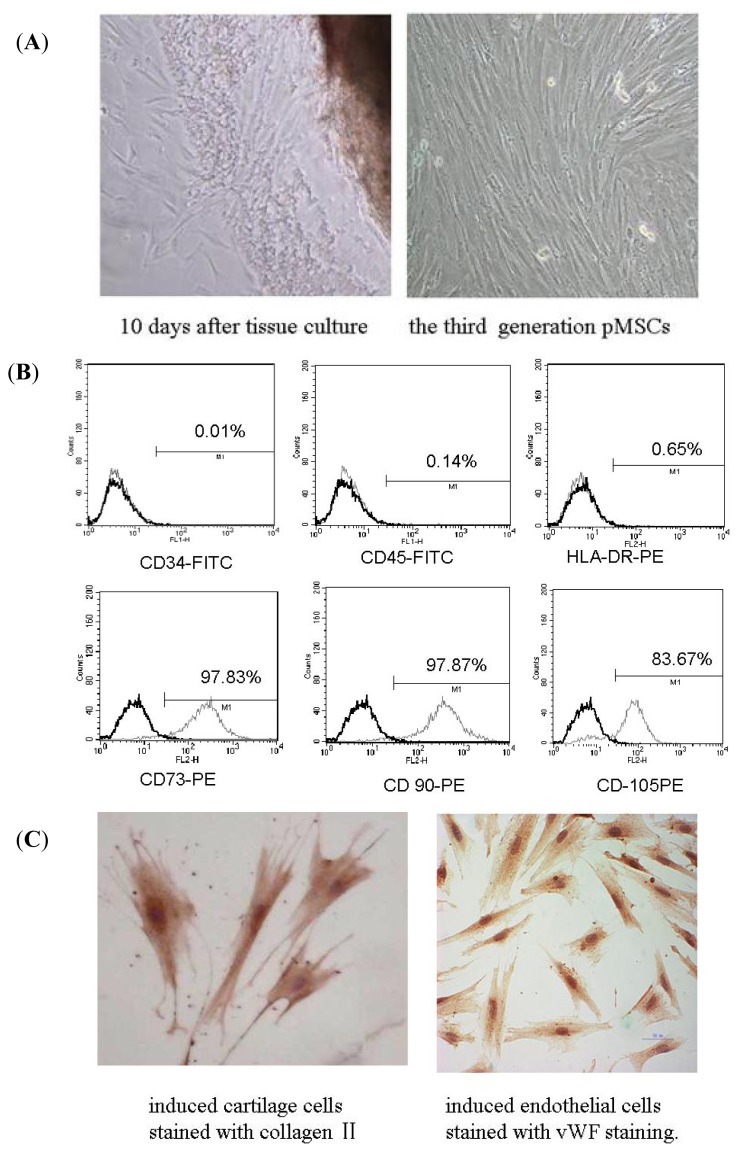
Isolation and identification of pMSCs. (**A**) Isolation of pMSCs by the method of tissue culture. Some cells appeared around tissues 10 days after culture. The third generation pMSCs had fibroblast-like morphology visualized by phase-contrast microscopy (100× magnification); (**B**) Flow cytometry assay of pMSCs: most of pMSCs were CD73, CD90 and CD105 positive; CD34, CD45 and HLA-DR negative; and (**C**) Differentiation ability of pMSCs: pMSCs can differentiate into cartilage cells and endothelial cells (400× magnification).

The pMSCs were isolated by the method of tissue culture. Some cells appeared around tissues after 10 days of culture (Figure 1A). Hematopoietic cells present were depleted during passaging. The third generation of pMSCs was morphologically defined by a fibroblast-like appearance and spiral-shaped growth (Figure 1A). The third passage cells were tested by flow cytometry. Most of them were positive for CD73, CD90 and CD105, a set of markers required for expression according to the minimal criteria for defining multipotent MSC adopted by the International Society for Cell Therapy (Figure 1B). They are also negative for CD34 (progenitors/endothelial cells), CD45 (leukocytes) and HLA-DR (Figure 1B). Each batch of pMSCs was further characterized by confirming their specific ability to differentiate into chondrocytes and endothelial cells (Figure 1C). Induced cartilage cells were stained with collagen II and induced endothelial cells were stained with von Willebrand factor (vWF). Only cells that met these criteria were used in subsequent experiments.

### 2.2. The pMSCs Hypoxia Culture Medium Is Better Protection for H_2_O_2_-treated-caco2 than pMSCs Normoxia Culture Medium

**Figure 2.**
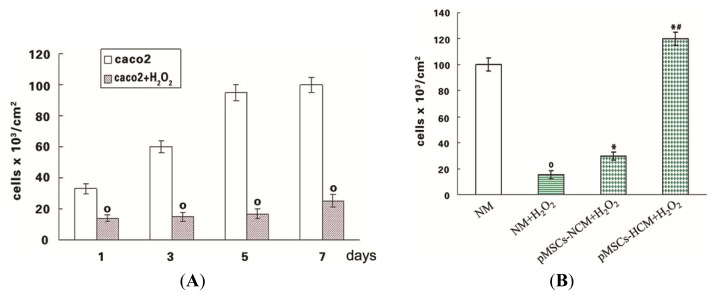
Protective effect of pMSCs-HCM on H_2_O_2_-treated-caco2 cells. (**A**) H_2_O_2_ exerted cytotoxic effect on caco2 cell viability. Caco2 cells were incubated with H_2_O_2_ (100 μM) for 12 h. After H_2_O_2_ removal, cells were maintained in normal medium and viable cells were counted after 1, 3, 5, 7 days; º *p* < 0.05 *versus* caco2 cells at the corresponding times (*n* = 6); and (**B**) pMSCs-HCM has a better protective effect for H_2_O_2_-treated-caco2 cells than that of pMSCs-NCM. Caco2 cells were incubated with 100 μM H_2_O_2_ for 12 h; after drug withdrawal, cells were cultured in normal medium, pMSCs-NCM, and pMSCs-HCM. Five days later, viable cells were counted by trypan blue staining; º *p* < 0.05 *versus* NM; * *p* < 0.05 *versus* NM + H_2_O_2_; ^#^ *p* < 0.05 *versus* pMSCs-NCM + H_2_O_2_ (*n* = 10).

In the *in vitro* model of H_2_O_2_ induced toxicity, caco2 cells were exposed to 100 μM H_2_O_2_ for 12 h, and then cultured in normal medium (NM). After the H_2_O_2_ withdrawal, viable cell counts were performed at various time intervals by trypan blue staining. Viable caco2 cells at 1 day after H_2_O_2_ significantly decreased, compared with the untreated cells (*p* < 0.05; Figure 2A). After five days, the number of viable caco2 cells was further significantly reduced 78% with respect to control (*p* < 0.05; Figure 2A). Additionally, a marked toxicity persisted at seven d (*p* < 0.05; Figure 2A).

The protective capability of pMSCs conditioned medium that included pMSCs normoxia culture medium (pMSCs-NCM) and pMSCs hypoxia culture medium (pMSCs-HCM) was investigated by culturing H_2_O_2_-treated-caco2 in them. Results showed that viable cell number of H_2_O_2_-treated-caco2 in both pMSCs-NCM and pMSCs-HCM significantly increased compared with that in normal medium (NM) five days later (*p* < 0.05; Figure 2B). However, pMSCs-HCM significantly increased the number of viable cells compared to pMSCs-NCM (*p* < 0.05; Figure 2B).

### 2.3. pMSCs Hypoxia Culture Medium Produces Enhanced Protection for H_2_O_2_-treated-caco2 Cells through Increased IGF-1

**Figure 3.**
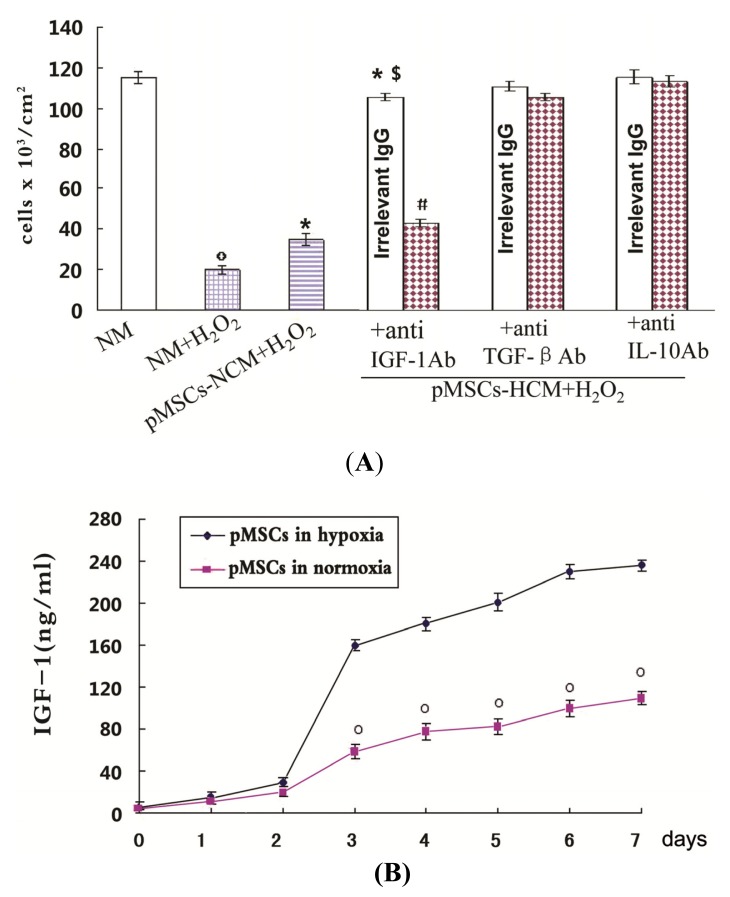

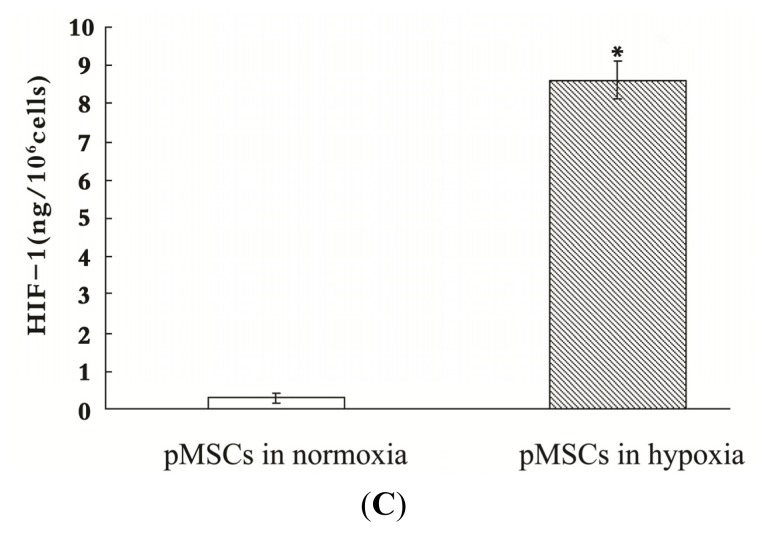
The pMSCs hypoxia culture mediums produce a better protective effect on caco2 cells from H_2_O_2_ through more IGF-1. (**A**) Effect of anti-IGF-1, anti-TGF-β and anti-IL-10 Ab on protective effect of pMSCs-HCM. Three hours after culturing H_2_O_2_-treated caco2 cells in pMSCs-HCM, cells were exposed to specific functional blocking Ab against IGF-1, TGFβ, L-10 or the corresponding irrelevant IgG. Cell count was evaluated after five days, º *p* < 0.05 *versus* NM; * *p* < 0.05 *versus* NM + H_2_O_2_; ^$^ *p* < 0.05 *versus* pMSCs-NCM + H_2_O_2_; ^#^ *p* < 0.05 *versus* corresponding irrelevant IgG. (*n* = 6); (**B**) Hypoxia induced more IGF-1 secreted by pMSCs. The secretion of IGF-1 by pMSCs in both normoxia and hypoxia increased with time. There was no difference in the early two days. From the third day on, the secretion by pMSCs cultured in hypoxia was much higher than that in normoxia, and the difference persisted at seven days, º *p* < 0.05 *versus* pMSCs in hypoxia; and (**C**) Activated HIF-1 of pMSCs in hypoxia or normoxia measured by HIF-1 ELISA. pMSCs were cultured in hypoxia or normoxia for seven days and then the amount of activated HIF-1 was detected. Figure 3C shows mean amounts of activated HIF-1 normalized to amounts per million harvested cells. pMSCs in hypoxia have much more activated HIF-1 than that in normoxia; * *p* < 0.05 *versus* pMSCs in normoxia.

The roles of IGF-1, TGF-β and IL-10 in the enhanced protective effects of pMSCs-HCM for H_2_O_2_-treated-caco2 cells were investigated. Both pMSCs-HCM with irrelevant antibody (Ab) and pMSCs-NCM made more H_2_O_2_-treated-caco2 cells alive than NM (*p* < 0.05; Figure 3A), and the former had more viable cells than the latter (*p* < 0.05; Figure 3A). In pMSCs-HCM, addition of specific Ab against IGF-1 led to a significant decrease in viable cell number compared with addition of irrelevant Ab (*p* < 0.05; Figure 3A). However, addition of Ab against TGF-β and IL-10 did not affect cell viability, as observed with irrelevant Ab. ELISA was used to detect the secretion of IGF-1 by pMSCs. Figure 3B showed that the secretion of IGF-1 by pMSCs increased with time under the condition of both normoxia and hypoxia. There was no obvious difference in the early 2 days, however from the third day on the concentration of IGF-1 in pMSC-HCM was significantly higher than that of pMSC-NCM (*p* < 0.05; Figure 3B). The difference still existed until the seventh day. We also detected the activation of HIF-1 to confirm the sensitivity of pMSCs to hypoxic conditions. We used the Trans-A method to measure HIF-1 activity of pMSCs in hypoxia and normoxia. This method only detected activated HIF-1 in nuclear extracts of cells. To confirm the specificity of the assay, we added either the wild-type or mutated consensus oligonucleotides to the tested samples. The addition of the wild-type nucleotide to the tested samples completely suppressed HIF-1 binding to the probe immobilized on the assay plate. The addition of the mutated oligonucleotide did not affect the results, so confirming the specificity of the testing method. We found that pMSCs in normoxia has very low levels of active HIF-1, but pMSCs in hypoxia have accumulated large amounts of these factors (*p* < 0.05; Figure 3C). What was more, results of the immunofluorescence test showed that caco2 cells stably expressed IGF-1R, either in normal medium or pMSCs conditioned medium, with or without H_2_O_2_ treatment (Supplemental Figure S1).

### 2.4. pMSCs Hypoxia Culture Medium Is More Conducive to Promotion of Proliferation of H_2_O_2_-treated-caco2 Cells than pMSCs Normoxia Culture Medium via IGF-1

**Figure 4.**
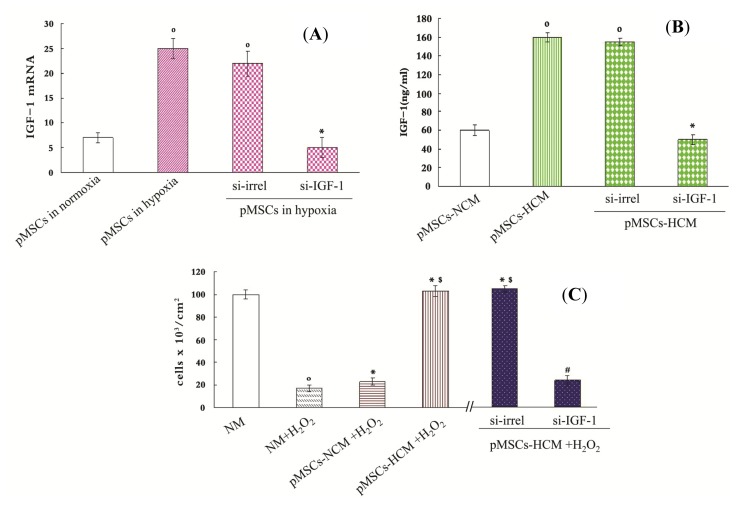

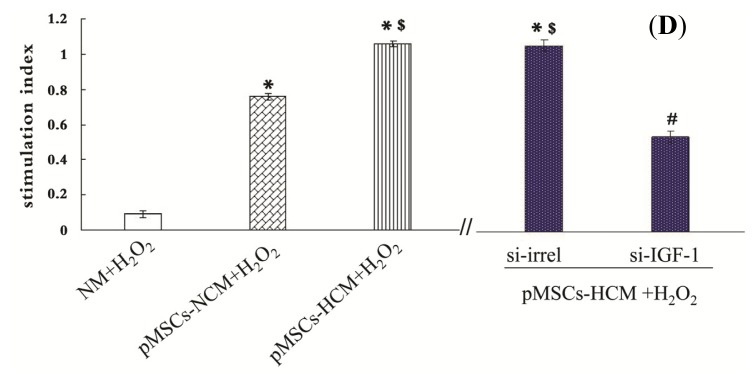
The pMSCs hypoxia culture medium is more conducive to promote H_2_O_2_-treated-caco2 cells proliferation via IGF-1. (**A** and **B**) Silence IGF-1 in pMSCs by siRNA: (**A**) After two-day culture, pMSCs in hypoxia had much more IGF-1 mRNA than that in normoxia, and it is the same with pMSCs in hypoxia transfected with si-irrel. When cultured in hypoxia, si-IGF-1-pMSCs had much less IGF-1 mRNA than si-irrel-pMSCs. º *p* < 0.05 *versus* pMSCs in normoxia; * *p* < 0.05 *versus* si-irrel-pMSCs in hypoxia (*n* = 6); (**B**) IGF-1 protein level in pMSCs-HCM and siirrel-pMSCs-HCM were much higher than that in pMSCs-NCM after four-day culture. IGF1 protein level in si-IGF-1-pMSCs-HCM obviously decreased compared with si-irrelpMSCs-HCM; º *p* < 0.05 *versus* pMSCs-NCM; * *p* < 0.05 *versus* si-irrel-pMSCs-HCM (*n* = 6); (**C**) Trypan blue staining is used to detect viable cell number. After H_2_O_2_-treated-caco2 cells were cultured in NM, pMSCs-NCM, pMSCs-HCM, si-irrel-pMSCs-HCM or si-IGF-1pMSCs-HCM for five days, viable cell number were assessed. º *p* < 0.05 *versus* NM; * *p* < 0.05 *versus* NM + H_2_O_2_; ^$^ *p* < 0.05 *versus* pMSCs-NCM + H_2_O_2_; ^#^ *p* < 0.05 *versus* si-irrel-pMSCs-HCM + H_2_O_2_ (*n* = 6); and (**D**) MTT is used to detect cellular metabolic activity. After H_2_O_2_-treated caco2 cells were cultured in NM, pMSCs-NCM, pMSCs-HCM, si-irrel-pMSCs-HCM or si-IGF-1pMSCs-HCM for five days, cellular metabolic activity were assessed by MTT. * *p* < 0.05 *versus* NM + H_2_O_2_; ^$^ *p* < 0.05 *versus* pMSCs-NCM + H_2_O_2_; ^#^ *p* < 0.05 *versus* si-irrel-pMSCsHCM + H_2_O_2_ (*n* = 6).

For assessing the direct role of IGF-1 in the protective effect of pMSCs-HCM, RNA interference technique to block *IGF-1* gene expression in pMSCs was used. We designed small interfering RNA (siRNA) for the two transcript variants of *IGF-1* (si-IGF-1). After two days of culture in hypoxia, the *IGF-1* mRNA level of pMSCs transfected with si-IGF-1 was inhibited by 76% with respect to that transfected with irrelevant siRNA (si-irrel, *p* < 0.05; Figure 4A), and it was the same with the results of ELISA. The pMSCs transfected with si-IGF-1 exhibited a marked reduction (63%) of IGF-1 protein as compared with that transfected with si-irrel after 4 days of culture in hypoxia (*p* < 0.05, Figure 4B).

We used trypan blue staining and MTT to demonstrate pMSCs-HCM is more conducive to promote the proliferation of H_2_O_2_-treated-caco2 than pMSCs-NCM via IGF-1. Trypan blue staining is used to detect viable cell number and MTT is an indicator of cellular metabolic activity. We cultured H_2_O_2_-treated-caco2 cells in NM, pMSCs-NCM, pMSCs-HCM, si-irrel-pMSCs-HCM and si-IGF-1-pMSCs-HCM. Results of trypan blue staining showed that viable cell number of H_2_O_2_-treated-caco2 cells in pMSCs-NCM, pMSCs-HCM and si-irrel-pMSCs-HCM increased compared with that in NM five days later (*p* < 0.05; Figure 4C). The pMSCs-HCM and si-irrel-pMSCs-HCM made much more cells alive than pMSCs-NCM (*p* < 0.05; Figure 4C). After knocking down expression of *IGF-1*, viable cell number in si-IGF-1-pMSCs-HCM significantly decreased (*p* < 0.05; Figure 4C).

We used MTT to detect the OD values of each group, and took the OD value of untreated caco2 cells in NM as control. OD values of H_2_O_2_-treated-caco2 cells in other medium were divided by control to get the stimulation index. Figure 4D showed that H_2_O_2_ can inhibit metabolic activity of caco2 cells. On the contrary, the pMSCs-NCM, pMSCs-HCM and si-irrel-pMSCs-HCM promoted the metabolic activity of H_2_O_2_-treated-caco2 cells (*p* < 0.05; Figure 4D). The effect of the latter two was stronger than that of the former (*p* < 0.05; Figure 4D). More IGF-1 secreted by hypoxic culture pMSCs was the reason for the better protective effect, which was documented by silencing IGF-1. Stimulation index in si-IGF-1-pMSCs-HCM significantly decreased compared with that in si-irrel-pMSCs-HCM (*p* < 0.05; Figure 4D).

### 2.5. pMSCs Hypoxia Culture Medium Has Greater Capacity of Limiting H_2_O_2_-treated-caco2 Apoptosis via Increased IGF-1 Compared to pMSCs Normoxia Culture Medium

**Figure 5.**
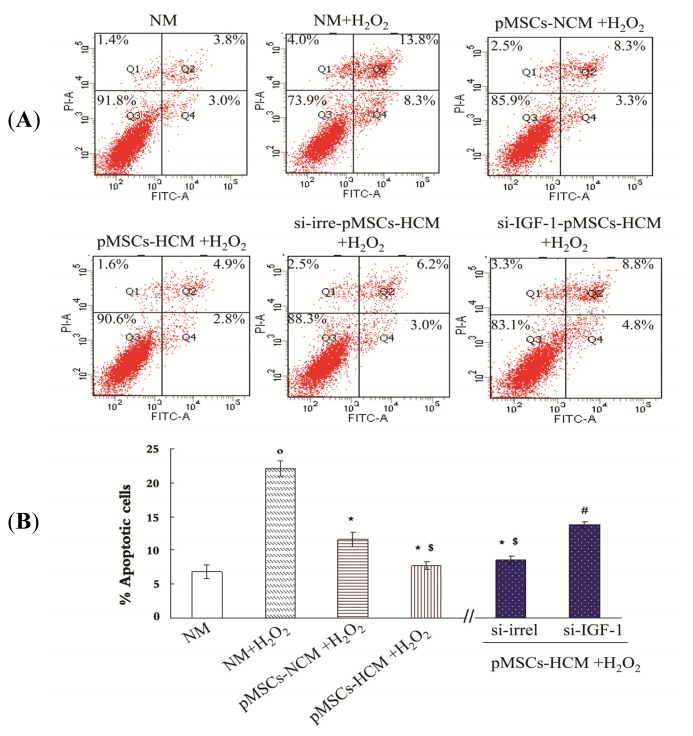
pMSCs hypoxia culture medium has stronger effect on limiting H_2_O_2_-treated-caco2 apoptosis via IGF-1 compared with pMSCs normoxia culture medium. (**A**) After five days culture, untreated caco2 cells in NM, H_2_O_2_-treated caco2 cells in NM, pMSCs-NCM, si-irrel-pMSCs-HCM or si-IGF-1-pMSCs-HCM, percentage of apoptotic cells (early apoptosis is FITC-positive, late apoptosis is FITC- and propidium iodide-postive) were analyzed by FACS; and (**B**) Data was expressed as the mean ± SEM of experiments repeated three times. º *p* < 0.05 *versus* NM. * *p* < 0.05 *versus* NM + H_2_O_2_; ^$^ *p* < 0.05 *versus* pMSCs-NCM + H_2_O_2_; ^#^ *p* < 0.05 *versus* si-irrel-pMSCs-HCM + H_2_O_2_ (*n* = 3).

An Annexin V-FITC apoptosis detection kit was used to detect apoptotic caco2 cells. As shown in Figure 5, regions 2 and 4 reflect apoptotic cells, including early apoptotic cells (region 4) and late apoptotic cells (region 2). Compared with untreated caco2 cells in NM, H_2_O_2_ can significantly promote apoptosis (*p* < 0.05; Figure 5B). The pMSCs-NCM and si-irrel-pMSCs-HCM played a protective role on H_2_O_2_-treated-caco2 cells compared with NM (*p* < 0.05; Figure 5B). The pMSCs-HCM and si-irrel-pMSCs-HCM were more capable of inhibiting cells apoptosis than pMSCs-NCM (*p* < 0.05; Figure 5B). The enhanced protection of si-irrel-pMSCs-HCM mainly depended on IGF-1. Apoptotic cells in si-IGF-1-pMSCs-HCM were much more than that in si-irrel-pMSCs-HCM (*p* < 0.05; Figure 5B).

### 2.6. Addition of IGF-1 Enhances the Protective Effect of pMSCs-NCM on H_2_O_2_-treated Caco2 Cells

**Figure 6.**
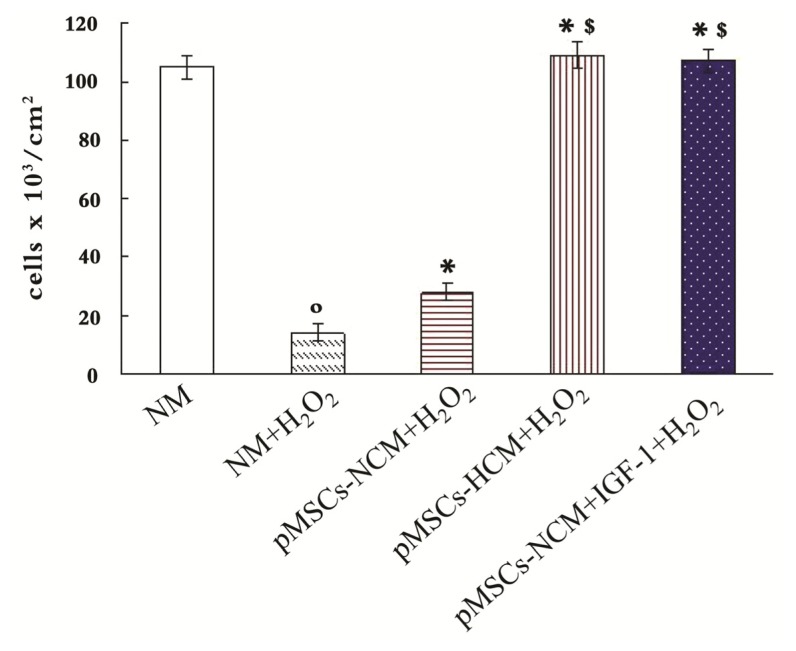
Addition of IGF-1 can enhance the protective effect of pMSCs-NCM on H_2_O_2_-treated-caco2 cells. The untreated caco2 in NM, H_2_O_2_-treated-caco2 cells in NM, pMSCs-NCM, pMSCs-HCM and pMSCs-NCM were cultured with IGF-1 (200 ng/mL). 5 days later, trypan blue staining was used to determine the viable cells. º *p* < 0.05 *versus* NM, * *p* < 0.05 *versus* NM + H_2_O_2_, and ^$^ *p* < 0.05 *versus* pMSCs-NCM + H_2_O_2_.

As can be seen from the Figure 6, after addition of IGF-1 (200 ng/mL) into the pMSCs-NCM, the protective effect of pMSCs-NCM on H_2_O_2_-treated caco2 cells significantly increased (*p* < 0.05).

### 2.7. Discussion

pMSCs are novel mesenchymal stem cells found in recent years. Because of their wide variety of sources, easy separation, rapid proliferation and low immunogenicity, they are considered as a good source of mesenchymal stem cells. Studies have shown that pMSCs had a certain therapeutic effect on myocardial infarction and bladder injury diseases [16,19]. Therapeutic effect has been attributed to the capacity of pMSC to induce tissue cells in injured sites to proliferate and differentiate into tissue cells. This latter phenomenon was limited in proportion and could not completely account for the observed reparative capacity of pMSCs. Therefore, a paracrine mechanism has been proposed as more suitable [27]. Current research on cell therapy in intestinal I/R injury however, mainly focus on bone marrow mesenchymal stem cells. If the pMSCs may play a therapeutic role on intestinal I/R injury, they would offer a better prospect for therapy.

Studies have shown that hypoxia can enhance paracrine effect of mesenchymal stem cells. Therefore, we speculate that protective effects of mesenchymal stem cells cultured in hypoxia on I/R injured intestinal epithelial cells may also be increased. This study therefore explored whether hypoxia can increase the capacity of pMSCs to protect I/R injured intestinal epithelial cells and the underlying mechanisms. In this investigation, we used caco2 cells to establish a model of I/R injured intestinal epithelial cells *in vitro*. Caco2 cells are the colon cancer cell line separated from human intestinal tissue and have the characteristics of epithelial cells. They are internationally recognized as the typical model for oral drug uptake experiments *in vitro*. Meanwhile, they can be transformed into intestinal epithelial cells and form monolayers, so they also are an ideal model system to study bacterial adhesion and invasion [28]. Intestinal epithelial cells can generate large amounts of oxygen free radicals after ischemia-reperfusion through xanthine oxidase. Oxygen radicals have the effect of cytotoxicity by lipid peroxidation and protein denaturation, a chain reaction that could expand and increase the damage. Therefore it is considered that reperfusion injury is mainly the result of a large number of oxygen free radicals. In our investigation, we used H_2_O_2_ to treat caco2 cells and found that viable cell numbers significantly decreased compared with untreated caco2 cells. This indicated that H_2_O_2_ was suitable for the model of I/R injured intestinal epithelial cells *in vitro*. The viable cell number of H_2_O_2_-treated-caco2 significantly increased when cultured in pMSCs conditioned medium (normoxia or hypoxia culture medium). Soluble protective cytokine in pMSCs conditioned medium could be responsible for the phenomenon because there was no direct contact of the pMSCs and caco2 cells in this process. We also found that pMSCs-HCM had better protection than pMSCs-NCM. This result suggested that hypoxia could enhance the protection of pMSCs through promoting their paracrine. This is consistent with the study of Yu, which reported that hypoxic preconditioning provided MSCs with stronger protection for acute ischemic renal injury [29].

pMSCs can secret a variety of cytokines [30–32]. To determine which of these cytokines is the key mediator that could account for the enhanced protection is a problem worthy of in-depth study. In our present study, we focused on IGF-1, which possesses mitogenic and anti-apoptotic properties [33,34]. TGF-β is involved in the occurrence and differentiation of intestinal epithelial cells [35]. IL-10 is the cytokine with anti-inflammatory activities [36]. Blocking of IGF-1 in pMSCs-HCM with a specific antibody resulted in a significant decrease in viable cell number of H_2_O_2_-treated-caco2 cells. Otherwise, incubation with Ab against TGF-β or IL-10 could not affect cell viability, as observed with irrelevant Ab. By using specific function blocking antibodies, a determinant role of IGF-1 in increased protective effect of pMSCs-HCM is confirmed.

There are two types of IGF, including IGF-1 and IGF-2. Many cells in our body can secret IGF-1 and circulating IGF-1 is primarily derived from the liver. IGF-1 exerts its biological functions through binding to specific receptors on the surface of target cells. It is relatively specific and different subtypes of IGF bind to their respective receptor. IGF-1 has the highest affinity with IGF-1R. Biological function of IGF-1 is so extensive that it can regulate the growth of body and development after birth, contribute to a variety of cell proliferation and inhibit apoptosis of follicular epithelial cells. Recent studies have shown that IGF-1 is an effective nutritional factor for the gastrointestinal tract. It can accelerate gastrointestinal damage repair by promoting the proliferation of mucosal cells [37].

To further confirm that IGF-1 can enhance the protection of pMSCs-HCM, we used ELISA to detect the content of IGF-1 in pMSCs culture supernatant. Results showed that pMSCs can secrete IGF-1, which is consistent with the reports of Togel [27] and Abboud [38]. We also found that IGF-1 levels in pMSCs-HCM were increased compared to that in pMSCs-NCM. In order to increase the secretion of IGF-1 in pMSCs, we should find the best concentration of oxygen and the best time for cell culture. The pMSCs were divided into three groups and respectively incubated in 1% oxygen incubator, 3% oxygen incubator and 5% oxygen incubator. In the first two days, the IGF-1 in the cell culture medium of the three groups had not changed. From the third day on, the IGF-1 of the three groups begun to increase significantly, and this increase continued until the seventh day. From the third day to the seventh day, the IGF-1 in both 1% O_2_ group and 3% O_2_ group were much more than that in the 5% O_2_ group. The IGF-1 in 1% O_2_ group was slightly more than that in 3% O_2_ group. Therefore, hypoxic preconditioning in this experiment was that culturing pMSCs in 1% O_2_ incubator for seven days (data not shown). We also detected the amount of activated HIF-1 in pMSCs cultured in both normoxia and hypoxia. Results show that pMSCs in hypoxia has much more activated HIF-1. Results of HIF-1 confirmed the sensitivity of pMSCs to hypoxic conditions. Immunofluorescence results showed caco2 cells stably expressed IGF-1 receptor, both in NM and pMSCs conditioned medium, with or without H_2_O_2_. Not only caco2 cells, but also normal intestinal epithelial cells express the IGF-1 receptor through which IGF-1 can promote proliferation as reported by Park [39].

Next, we investigated the mechanism of protection. RNAi technology was used to reduce expression of IGF-1 in hypoxic cultured pMSCs at both the mRNA and protein level. MTT assay and trypan blue staining were used to detect proliferation of cells. Trypan blue staining is used to detect viable cell number and MTT is an indicator of cellular metabolic activity. Results of trypan blue staining showed that viable cell number of H_2_O_2_-treated-caco2 in pMSCs-NCM, pMSCs-HCM and si-irrel-pMSCs-HCM increased compared with that in NM after five days of culture. pMSCs-HCM and si-irrel-pMSCs-HCM significantly increased the number of viable cells compared to pMSCs-NCM. After knocking down expression of IGF-1, viable cell number in si-IGF-1-pMSCs-HCM decreased significantly.

We used MTT to detect OD values of the cells. The OD value of untreated caco2 cells in NM was taken as control, OD values of H_2_O_2_-treated-caco2 cells in other medium are divided by control to get the stimulation index. The results were similar with that of trypan blue staining. The metabolic activity of H_2_O_2_-treated-caco2 cells in pMSC-NCM, pMSCs-HCM and si-irrel-pMSC-HCM are better than that in NM. When pMSCs were cultured in hypoxia, transfection with si-IGF-1 led to much less metabolic activity in pMSC-HCM than transfection with si-irrel. The results of MTT assay and trypan blue staining confirmed that the increased pro-proliferation ability of pMSC-HCM might be mediated by IGF-1.

This study also established an anti-apoptotic activity of IGF-1. In our experiment, H_2_O_2_ induced consistent apoptosis of caco2 cell, which was reduced by pMSCs-NCM, pMSCs-HCM and si-irrel-pMSCs-HCM, and the effects of pMSCs-HCM and si-irrel-pMSCs-HCM were stronger. Data showed that silencing IGF-1 resulted in increased percentage of apoptotic cells in pMSCs-HCM, and this suggested that IGF-1 was the key cytokine for the decreased number of apoptotic cells in pMSCs-HCM. Addition of IGF-1 can enhance the viable cell number of H_2_O_2_-treated-caco2 cell in pMSCs-NCM, which also demonstrated that IGF-1 is the key cytokine for the enhanced protective effect of pMSCs in hypoxia.

Our experiments showed that hypoxia could enhance the protective effect of pMSCs on I/R injured intestinal epithelial cells. This result is similar to previous findings that hypoxia can enhance the therapeutic effect of mesenchymal stem cells in animal models of renal I/R injury [29]. The mechanism involves promoting paracrine action and improving anti-apoptotic ability of mesenchymal stem cells [24,25]. Our work also found that hypoxia makes pMSCs grow faster, show a more uniform shape of long spindle, secrete significantly more protective cytokines, and have stronger anti-apoptotic capacity. Therefore, we speculate that hypoxia is an effective method to improve therapeutic effect of pMSCs on intestinal I/R injury.

## 3. Experimental Section

### 3.1. Isolation and Passage of Placenta-Derived Mesenchymal Stem Cells

After maternal consent, placental tissue of cesarean full-term healthy newborns was taken under sterile conditions. pMSCs were isolated as previously described [40]. Cells were cultured in DMEM with high glucose content (Invitrogen, Carlsbad, CA, USA) supplemented with 10% of fetal bovine serum (Bioind, Mazkeret Batya, Israel), 1% penicillin/streptomycin (Gibco, Grand Island, NY, USA), 0.1 mM β-mercaptoethanol (Sigma, St. Louis, MO, USA) and 2 mM l-glutamine (Invitrogen, Carlsbad, CA, USA). When cells reached 90% of confluence, they were harvested by trypsinization (Invitrogen, Carlsbad, CA, USA) and reseeded in a new dishes at a density of 5 × 10^5^ per dish (100-mm). All experiments were performed using cells at passage three.

### 3.2. Trypan Blue Staining

Cells were trypsinized, and PBS was used to make appropriately diluted single cell suspensions. Single cell suspension were mixed with trypan blue solution (2×) in a ratio of 1:1. Under the light microscope live and dead cells were respectively counted within three minutes. Dead cells are stained blue, living cells are colorless and transparent.

### 3.3. Cartilage Cells Differentiation and Collagen II Staining

For cartilage cells differentiation, the media contained high-glucose DMEM (4.5 g/mL) supplemented with 10 mol/L dexamethasone (Sigma, St. Louis, MO, USA), 10 ng/mL recombinant human transforming growth factor-β1 (R&D, Valais, Switzerland), 6.25 μg/mL insulin (Sigma, St. Louis, MO, USA), 6.25 μg/mL transferin (Sigma, St. Louis, MO, USA), 50 mg/mL vitamin C (Sigma, St. Louis, MO, USA), 100 ng/mL insulin-like growth factor (Biovision, Milpitas, CA, USA), and 5% FBS or 5% CBS, l-DMEM medium. Differentiation medium was replaced every three days. After three weeks, the cells were fixed with 4% formalin and immunocytochemistry was carried out using mouse anti-human collagen (1:100; Boster, Wuhan, China) according to the instructions of manufacturer.

### 3.4. Endothelial Cells Differentiation and von Willebrand Factor Staining

For endothelial cell differentiation, the media contained 50 ng/mL recombinant human vascular endothelial growth factor (rHuVEGF, Sigma, St. Louis, MO, USA), 10 ng/mL basic fibroblast growth factor (rHubFGF, Sigma, St. Louis, MO, USA), 0.1mM β-mercaptoethanol (Sigma, St. Louis, MO, USA), 2 mM l-glutamine (Invitrogen, Carlsbad, CA, USA), 0.1 mM nonessential amino acids (MEM), and 1% penicillin/streptomycin, and 5% FBS or CBS, l-DMEM medium. Differentiation medium was replaced every three days. At day eight, the cells were fixed with 4% formalin and immunocytochemistry was carried out using mouse anti-human vWF (1:100, Maxim, Fuzhou, China) according to the instructions of manufacturer.

### 3.5. Flow Cytometry to Detect Cell Surface Markers and Apoptosis

Cells were trypsinized, washed twice in PBS and centrifuged at 1000 rpm for 10 min. Cells were resuspended in PBS in a final concentration of 5 × 10^5^/mL and incubated for 30 min at 4 °C with antibody against cell surface markers: CD29-FITC, CD44-FITC, CD31-FITC, CD34-APC, CD45-FITC, HLA-DR-FITC (all from B&D, Franklin Lakes, NJ, USA). After incubation cells were washed with PBS, centrifuged for 5 min at 2000 rpm, resuspended and analyzed on a flow cytometer FACSCalibur after being filtered with screen cloth (B&D, Franklin Lakes, NJ, USA).

Flow cytometric analysis of apoptosis with Annexin-V/PI double staining (Beyotime, Haimen, China). After treatment with H_2_O_2_ (100 μM, 12 h), caco2 cells were cultured in normal medium or pMSCs conditioned medium for five days, about 3 × 10^5^ cells were harvested and washed twice with PBS, resuspended in 195 μL AnnexinV-FITC binding buffer. A volume of 5 μL Annexin V-FITC was added and mixed gently, and caco2 cells were stained in the dark for 10 min at room temperature. They were then centrifuged at 1000 rpm for 5 min and gently resuspended in 190 μL of Annexin V-FITC binding buffer. At last, 10 μL propidium iodide staining solution was added and gently mixed. The cells were kept on ice in the dark and immediately subjected to flow cytometry analysis after being filtered with screen cloth. Software FCM Cell Quest and Macquit were used to analyze the data (B&D, Franklin Lakes, NJ, USA).

### 3.6. ELISA

The evaluation of IGF-1 concentrations in cell culture supernatants was performed by ELISA. Cell culture supernatants were collected at different time points after hypoxia. IGF-1 was detected using IGF-1 ELISA kits (B&D, Franklin Lakes, NJ, USA) according to the instructions of manufacture. Concentration was calculated by regression analysis of a standard curve.

### 3.7. RNA Interference

The siRNA was selected in a region of the human IGF-1 transcript variants (NG-011713.1; NM-001111283 and NM-001111284). The si-IGF-1 and si-irrel RNA synthesized by Ambion (Austin, TX, USA). siRNA (100 pmol) were introduced in pMSCs that were cultured in six-well plates for 4 h by using siPORT amine transfection reagent (Ambion, Austin, TX, USA) according to the instruction of manufacturer. Forty-eight hours after transfection, the medium was changed and cells were cultured in hypoxic conditions (1% O_2_) for about 72 h, then the pMSCs conditoned medium for the subsequent experiments was collected.

### 3.8. RT-PCR and Quantitative Real-Time RT-PCR

RNA extraction and reverse-transcription reactions were carried out as previously described [41]. Quantitative real-time PCR was performed by a modified protocol [42]. The following primers were used for PCR amplification of human IGF-1 cDNA, sense CTGGAGTTGGTAGATTGCTGTTG, antisense CTTGAGAGGCAGGGACTAAGAT. All measurements were performed in triplicate. The average cycle threshold for cells was ~38 and for the no RNA control condition was >40 cycles.

### 3.9. Adding the Blocking Antibodies for IGF-1, TGFβ and IL-10

H_2_O_2_-treated-caco2 cells were cultured in pMSCs-HCM for three hours, and a kind of antibody or a respective irrelevant IgG was then added into the pMSCs-HCM. Five days later, the cell count is evaluated to find which one is the key cytokine for the protective effect of pMSCs-HCM. Antibodies include anti-IGF-1 Ab (0.25 μg/mL, R&D systems, Minneapolis, MN, USA), anti-TGF-βAb (20 μg/mL, Genzyme Corp., Cambridge, MA, USA), anti-IL-10 Ab (10 μg/mL, R&D systems, Minneapolis, MN, USA). These antibodies have been marked so that they can be added into culture medium and act as blocking antibodies [6].

### 3.10. MTT Determination of Cell Viability

The effect of pMSCs conditioned medium on proliferation of caco2 cells was evaluated by MTT assay. Caco2 cells were seeded into 96-well plates at the density of 12.5 × 10^3^/cm^2^ in six repeats and allowed to adhere over-night. After treatment with H_2_O_2_ (100 μM, 12 h), caco2 cells were cultured in normal medium or pMSCs conditioned medium for five days. 100 μL MTT (0.5 mg/mL) was added and the cells were incubated for 4 h. After medium was removed, 100 μL 20% SDS (containing 50% dimethylformamide) was added and incubated for another 24 h. Optical density values (OD) were measured at 570 nm wavelength with a microplate reader (Bio-Tek Cor-poration, Bennington, VT, USA). The experiments were repeated three times.

### 3.11. Cell Immunofluorescence

Immunofluorescence was used to detect IGF-1R in caco2 cells. Caco2 cells were seeded on glass coverslips. After 24 h some of them was treated with H_2_O_2_ (100 μM, 12 h) and then cultured in NM or pMSCs conditioned medium. Five days later, all the cells (untreated and H_2_O_2_-treated-caco2 cells) were fixed for 20 min at room temperature with 1% paraformaldehyde. After blocking in PBS containing 5% BSA, cells were labeled with anti-IGF-1R primary Abs at 4 °C overnight. Cells were washed three times with PBS and subsequently incubated with the indicated fluorophore-conjugated secondary antibodies for 1 h. Next, cells were labeled with DAPI for nuclear staining and coverslips were mounted on glass slides using prolong antifade mounting medium (Invitrogen). Cells were examined using fluorescence microscopy. Images were acquired by sequential excitation at 488 and 360 nm laser lines. Instrument settings were kept constant for each replicate.

### 3.12. Hypoxia Inducible Factor-1 Activity Assay

We use Thermo Fisher 311 anaerobic incubator (Thermo, Waltham, MA, USA) to control the concentration of oxygen (1%). Hypoxia setup is 1% O_2_, 5% CO_2_, and 94% N_2_. Passage 2 cells were seeded at 2000 cells/cm^2^ and cultured for seven days in either hypoxia or normoxia with intermittent media change. When the cultures reached approximately 70% confluence they were harvested and re-plated at the same concentration until reaching a confluence of 70%–80%. After that time, cells were immediately put on ice and the nuclear extracts were collected by nuclear extract kit (Active Motif Company, Carlsbad, CA, USA) according to the protocol of manufacturer. To determine activated hypoxia inducible factor levels, a commercially available ELISA kit (Active Motif Company, Carlsbad, CA, USA) was utilized on the nuclear extracts according to the manufactures instruction. The results were normalized to the number of cells.

### 3.13. Statistical Analysis

All experiments were repeated three times. Results are expressed as mean ± SEM. Significance of tests were performed using ANOVA and LSD post hoc with values of *p* < 0.05 considered significant. All other data were processed using SPSS.11 for Windows (SPSS Inc., Chicago, IL, USA).

## 4. Conclusions

In conclusion, our experimental results showed that pMSCs have certain protective effects on I/R injured intestinal epithelial cells. Hypoxia can enhance the protective effect of pMSCs by promoting the secretion of IGF-1. This highlights the therapeutic potential of hypoxic culture pMSCs in intestinal I/R injury. We intend to establish an animal model of intestinal I/R injury to further investigate this finding through *in vivo* experiments.

## Supplementary Information
