# Supplementary material for: Hypoxia Enhances Protective Effect of Placental-Derived Mesenchymal Stem Cells on Damaged Intestinal Epithelial Cells by Promoting Secretion of Insulin-Like Growth Factor-1
Source: Int J Mol Sci. 2014 Jan 27;15(2):1983–2002. doi: 10.3390/ijms15021983 (PMC3958833; doi:10.3390/ijms15021983)
Supplement: Supplementary file 1 [file ijms-15-01983-s001.pdf]

Supplementary Information

Figure S1. Detection of IGF-1 receptor on pMSCs. Untreated caco2 cultured in normal medium (NM), H_2O_2-treated-caco2 cultured in NM, pMSCs normoxia culture medium (pMSCs-NCM) or pMSCs hypoxia culture medium (pMSCs-HCM), all the cells were detected for expression of IGF-1 receptor by immunofluorescence. All of them were positive.

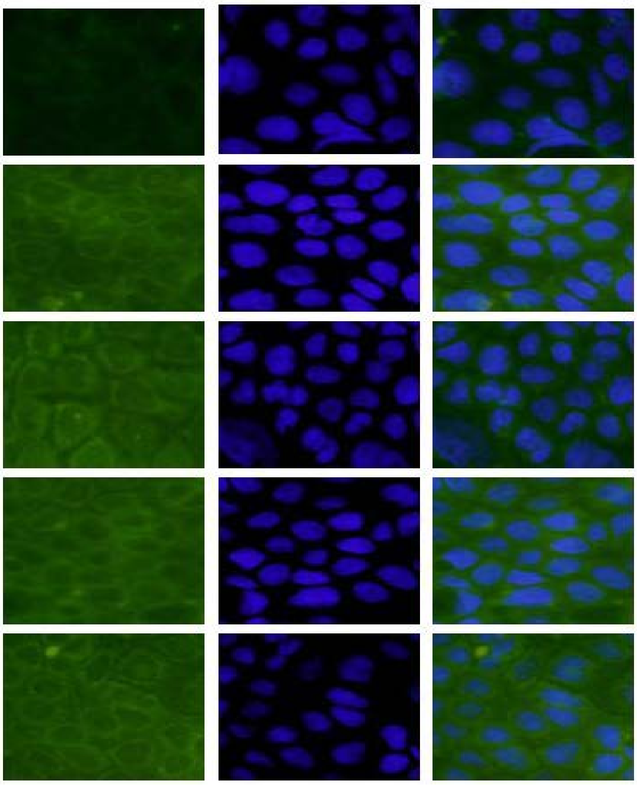

negative control

NM

NM + H_2O_2

pMSCs-NCM + H_2O_2

pMSCs-HCM + H_2O_2

© 2014 by the authors; licensee MDPI, Basel, Switzerland. This article is an open access article distributed under the terms and conditions of the Creative Commons Attribution license (http://creativecommons.org/licenses/by/3.0/).
